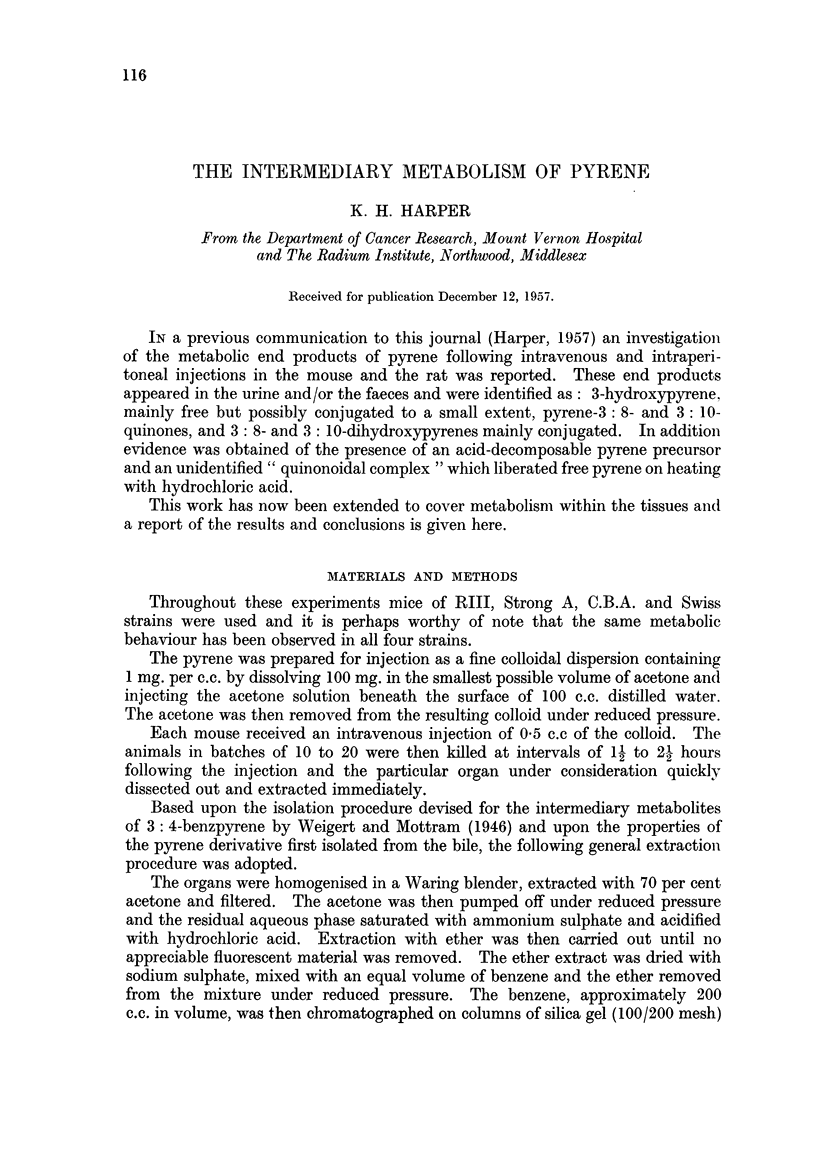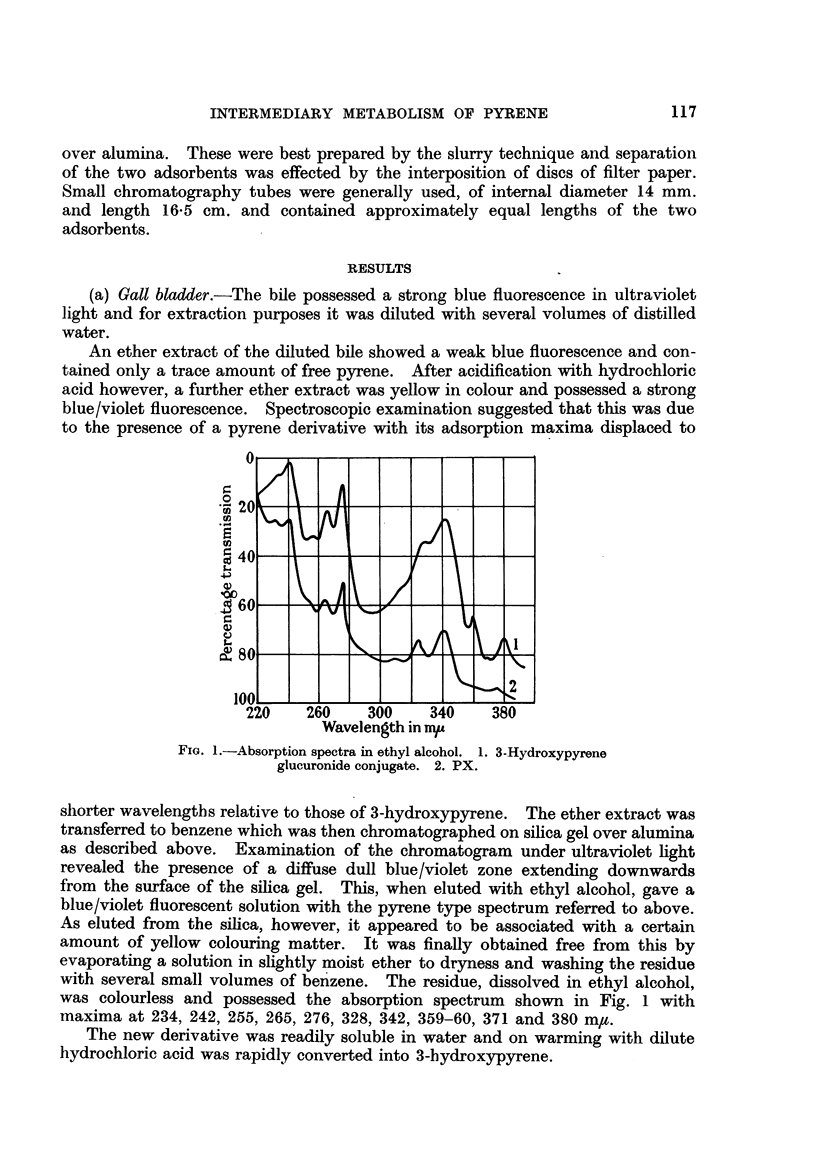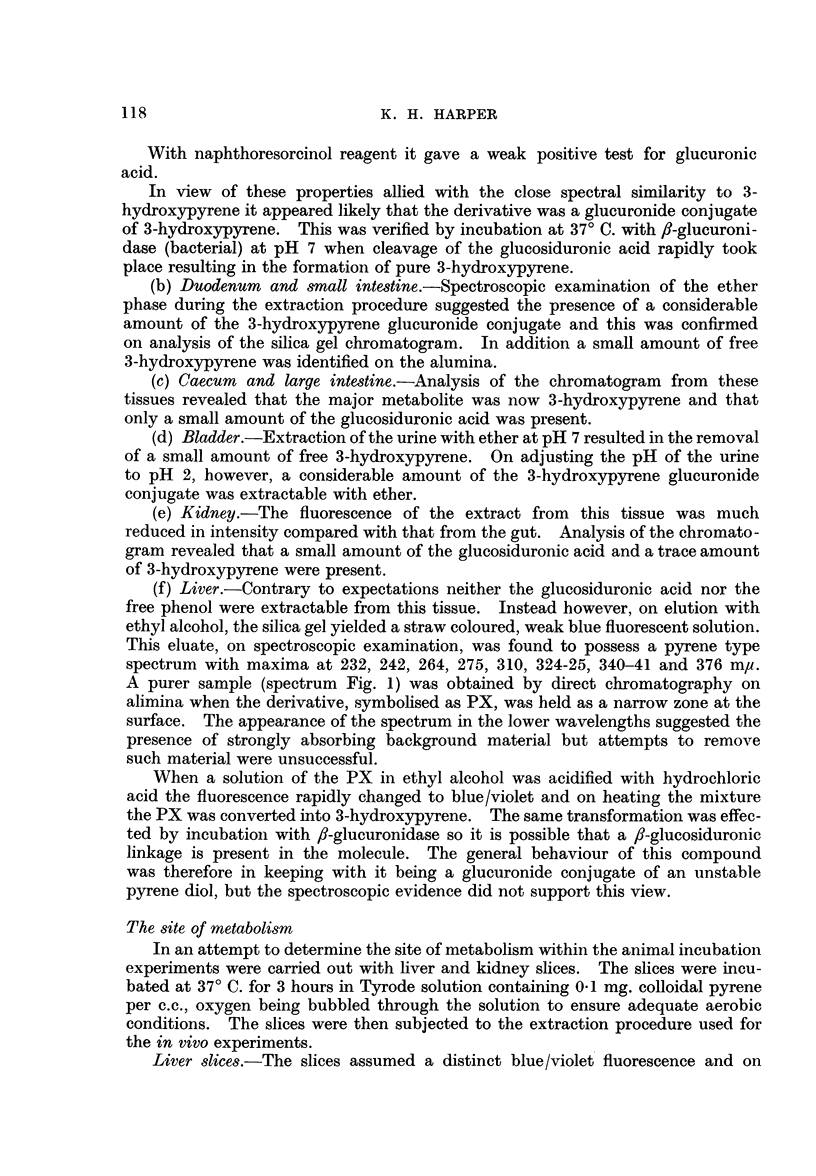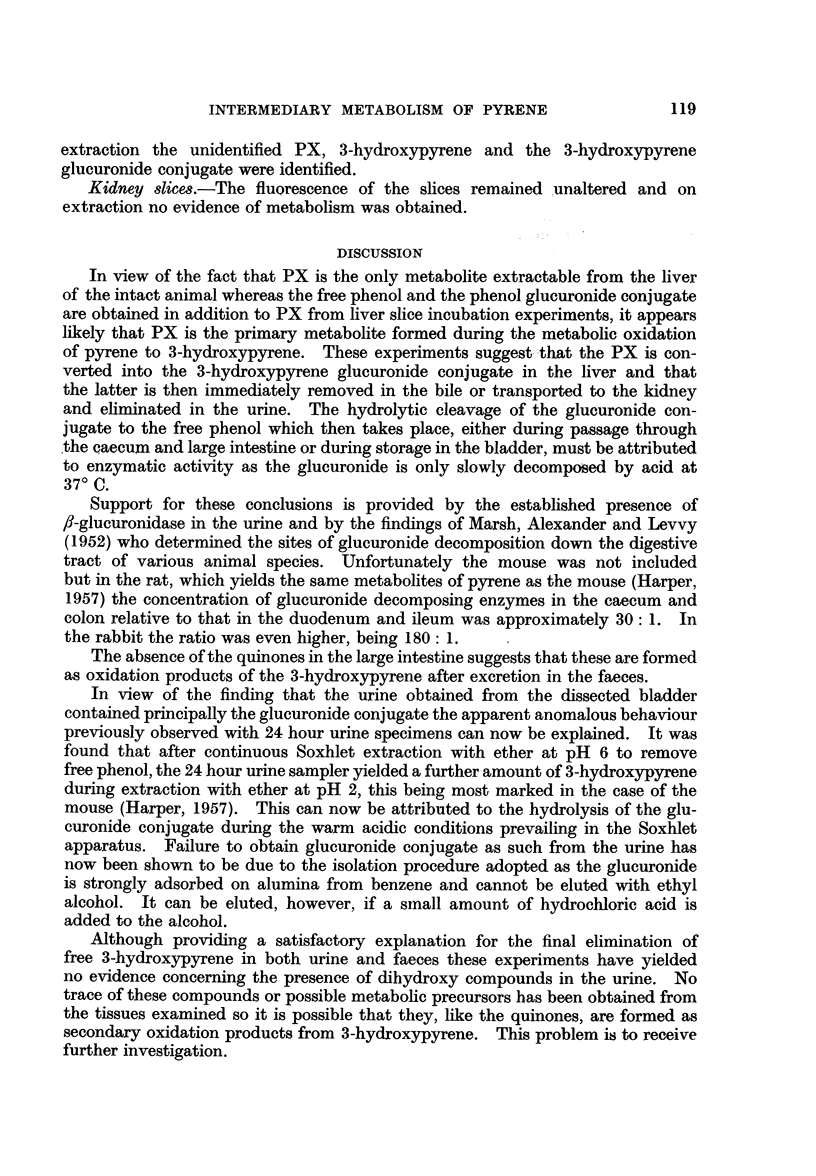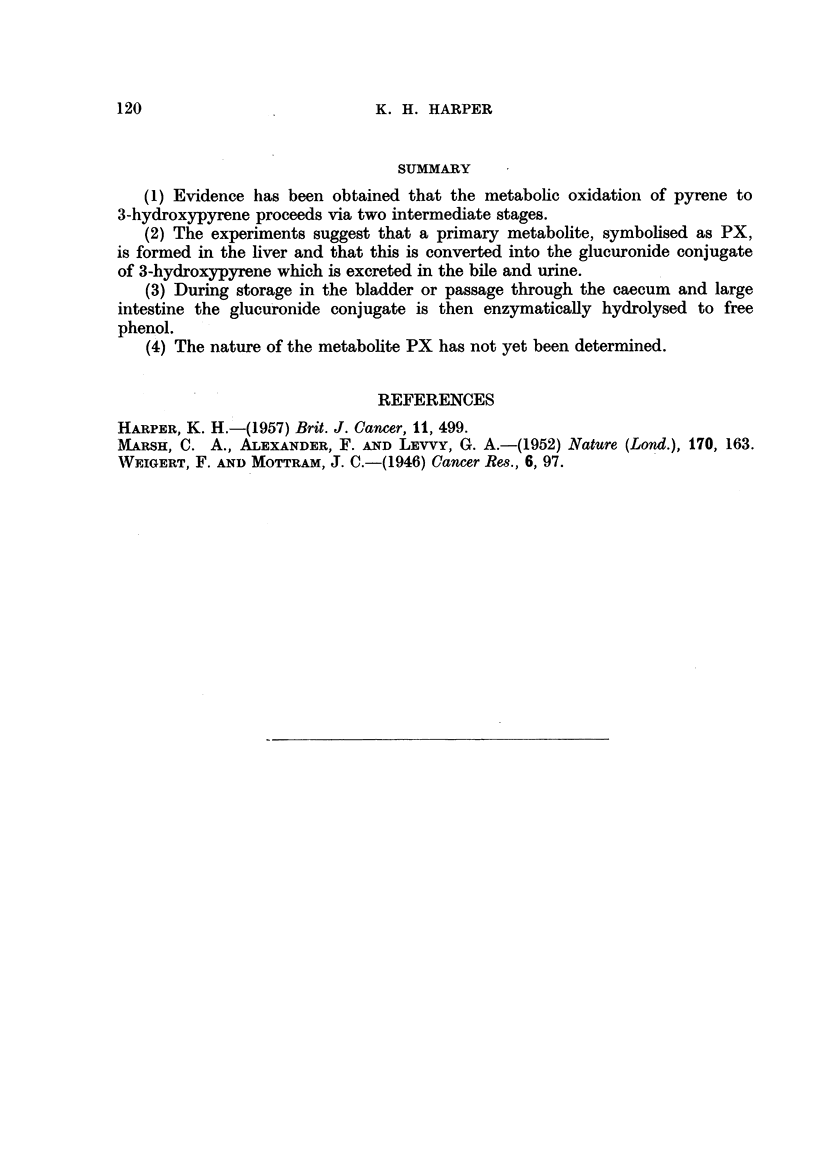# The Intermediary Metabolism of Pyrene

**DOI:** 10.1038/bjc.1958.15

**Published:** 1958-03

**Authors:** K. H. Harper


### THE INTERMEDIARY METABOLISM OF PYRENE

K. H. HARPER

*From the Department of Cancer Research, Mount Vernon Hospital and The Radium Institute, Northwood, Middlesex*

Received for publication December 12, 1957.

IN a previous communication to this journal (Harper, 1957) an investigation of the metabolic end products of pyrene following intravenous and intraperitoneal injections in the mouse and the rat was reported. These end products appeared in the urine and/or the faeces and were identified as : 3-hydroxypyrene, mainly free but possibly conjugated to a small extent, pyrene-3 : 8- and 3 : 10-quinones, and 3 : 8- and 3 : 10-dihydroxypyrenes mainly conjugated. In addition evidence was obtained of the presence of an acid-decomposable pyrene precursor and an unidentified " quinonoidal complex " which liberated free pyrene on heating with hydrochloric acid.

This work has now been extended to cover metabolism within the tissues and a report of the results and conclusions is given here.

### MATERIALS AND METHODS

Throughout these experiments mice of RIII, Strong A, C.B.A. and Swiss strains were used and it is perhaps worthy of note that the same metabolic behaviour has been observed in all four strains.

The pyrene was prepared for injection as a fine colloidal dispersion containing 1 mg. per c.c. by dissolving 100 mg. in the smallest possible volume of acetone and injecting the acetone solution beneath the surface of 100 c.c. distilled water. The acetone was then removed from the resulting colloid under reduced pressure.

Each mouse received an intravenous injection of 0·5 c.c of the colloid. The animals in batches of 10 to 20 were then killed at intervals of $1\frac{1}{2}$ to $2\frac{1}{2}$ hours following the injection and the particular organ under consideration quickly dissected out and extracted immediately.

Based upon the isolation procedure devised for the intermediary metabolites of 3 : 4-benzpyrene by Weigert and Mottram (1946) and upon the properties of the pyrene derivative first isolated from the bile, the following general extraction procedure was adopted.

The organs were homogenised in a Waring blender, extracted with 70 per cent acetone and filtered. The acetone was then pumped off under reduced pressure and the residual aqueous phase saturated with ammonium sulphate and acidified with hydrochloric acid. Extraction with ether was then carried out until no appreciable fluorescent material was removed. The ether extract was dried with sodium sulphate, mixed with an equal volume of benzene and the ether removed from the mixture under reduced pressure. The benzene, approximately 200 c.c. in volume, was then chromatographed on columns of silica gel (100/200 mesh)

over alumina. These were best prepared by the slurry technique and separation of the two adsorbents was effected by the interposition of discs of filter paper. Small chromatography tubes were generally used, of internal diameter 14 mm. and length 16·5 cm. and contained approximately equal lengths of the two adsorbents.

### RESULTS

(a) *Gall bladder.*—The bile possessed a strong blue fluorescence in ultraviolet light and for extraction purposes it was diluted with several volumes of distilled water.

An ether extract of the diluted bile showed a weak blue fluorescence and contained only a trace amount of free pyrene. After acidification with hydrochloric acid however, a further ether extract was yellow in colour and possessed a strong blue/violet fluorescence. Spectroscopic examination suggested that this was due to the presence of a pyrene derivative with its adsorption maxima displaced to

FIG. 1.—Absorption spectra in ethyl alcohol. 1. 3-Hydroxypyrene glucuronide conjugate. 2. PX.

shorter wavelengths relative to those of 3-hydroxypyrene. The ether extract was transferred to benzene which was then chromatographed on silica gel over alumina as described above. Examination of the chromatogram under ultraviolet light revealed the presence of a diffuse dull blue/violet zone extending downwards from the surface of the silica gel. This, when eluted with ethyl alcohol, gave a blue/violet fluorescent solution with the pyrene type spectrum referred to above. As eluted from the silica, however, it appeared to be associated with a certain amount of yellow colouring matter. It was finally obtained free from this by evaporating a solution in slightly moist ether to dryness and washing the residue with several small volumes of benzene. The residue, dissolved in ethyl alcohol, was colourless and possessed the absorption spectrum shown in Fig. 1 with maxima at 234, 242, 255, 265, 276, 328, 342, 359–60, 371 and 380 mμ.

The new derivative was readily soluble in water and on warming with dilute hydrochloric acid was rapidly converted into 3-hydroxypyrene.

With naphthoresorcinol reagent it gave a weak positive test for glucuronic acid.

In view of these properties allied with the close spectral similarity to 3-hydroxypyrene it appeared likely that the derivative was a glucuronide conjugate of 3-hydroxypyrene. This was verified by incubation at 37° C. with $\beta$-glucuronidase (bacterial) at pH 7 when cleavage of the glucosiduronic acid rapidly took place resulting in the formation of pure 3-hydroxypyrene.

(b) *Duodenum and small intestine.*—Spectroscopic examination of the ether phase during the extraction procedure suggested the presence of a considerable amount of the 3-hydroxypyrene glucuronide conjugate and this was confirmed on analysis of the silica gel chromatogram. In addition a small amount of free 3-hydroxypyrene was identified on the alumina.

(c) *Caecum and large intestine.*—Analysis of the chromatogram from these tissues revealed that the major metabolite was now 3-hydroxypyrene and that only a small amount of the glucosiduronic acid was present.

(d) *Bladder.*—Extraction of the urine with ether at pH 7 resulted in the removal of a small amount of free 3-hydroxypyrene. On adjusting the pH of the urine to pH 2, however, a considerable amount of the 3-hydroxypyrene glucuronide conjugate was extractable with ether.

(e) *Kidney.*—The fluorescence of the extract from this tissue was much reduced in intensity compared with that from the gut. Analysis of the chromatogram revealed that a small amount of the glucosiduronic acid and a trace amount of 3-hydroxypyrene were present.

(f) *Liver.*—Contrary to expectations neither the glucosiduronic acid nor the free phenol were extractable from this tissue. Instead however, on elution with ethyl alcohol, the silica gel yielded a straw coloured, weak blue fluorescent solution. This eluate, on spectroscopic examination, was found to possess a pyrene type spectrum with maxima at 232, 242, 264, 275, 310, 324-25, 340–41 and 376 m$\mu$. A purer sample (spectrum Fig. 1) was obtained by direct chromatography on alimina when the derivative, symbolised as PX, was held as a narrow zone at the surface. The appearance of the spectrum in the lower wavelengths suggested the presence of strongly absorbing background material but attempts to remove such material were unsuccessful.

When a solution of the PX in ethyl alcohol was acidified with hydrochloric acid the fluorescence rapidly changed to blue/violet and on heating the mixture the PX was converted into 3-hydroxypyrene. The same transformation was effected by incubation with $\beta$-glucuronidase so it is possible that a $\beta$-glucosiduronic linkage is present in the molecule. The general behaviour of this compound was therefore in keeping with it being a glucuronide conjugate of an unstable pyrene diol, but the spectroscopic evidence did not support this view.

*The site of metabolism*

In an attempt to determine the site of metabolism within the animal incubation experiments were carried out with liver and kidney slices. The slices were incubated at 37° C. for 3 hours in Tyrode solution containing 0·1 mg. colloidal pyrene per c.c., oxygen being bubbled through the solution to ensure adequate aerobic conditions. The slices were then subjected to the extraction procedure used for the *in vivo* experiments.

*Liver slices.*—The slices assumed a distinct blue/violet fluorescence and on extraction the unidentified PX, 3-hydroxypyrene and the 3-hydroxypyrene glucuronide conjugate were identified.

*Kidney slices.*—The fluorescence of the slices remained unaltered and on extraction no evidence of metabolism was obtained.

### DISCUSSION

In view of the fact that PX is the only metabolite extractable from the liver of the intact animal whereas the free phenol and the phenol glucuronide conjugate are obtained in addition to PX from liver slice incubation experiments, it appears likely that PX is the primary metabolite formed during the metabolic oxidation of pyrene to 3-hydroxypyrene. These experiments suggest that the PX is converted into the 3-hydroxypyrene glucuronide conjugate in the liver and that the latter is then immediately removed in the bile or transported to the kidney and eliminated in the urine. The hydrolytic cleavage of the glucuronide conjugate to the free phenol which then takes place, either during passage through the caecum and large intestine or during storage in the bladder, must be attributed to enzymatic activity as the glucuronide is only slowly decomposed by acid at 37° C.

Support for these conclusions is provided by the established presence of $\beta$-glucuronidase in the urine and by the findings of Marsh, Alexander and Levvy (1952) who determined the sites of glucuronide decomposition down the digestive tract of various animal species. Unfortunately the mouse was not included but in the rat, which yields the same metabolites of pyrene as the mouse (Harper, 1957) the concentration of glucuronide decomposing enzymes in the caecum and colon relative to that in the duodenum and ileum was approximately 30 : 1. In the rabbit the ratio was even higher, being 180 : 1.

The absence of the quinones in the large intestine suggests that these are formed as oxidation products of the 3-hydroxypyrene after excretion in the faeces.

In view of the finding that the urine obtained from the dissected bladder contained principally the glucuronide conjugate the apparent anomalous behaviour previously observed with 24 hour urine specimens can now be explained. It was found that after continuous Soxhlet extraction with ether at pH 6 to remove free phenol, the 24 hour urine sampler yielded a further amount of 3-hydroxypyrene during extraction with ether at pH 2, this being most marked in the case of the mouse (Harper, 1957). This can now be attributed to the hydrolysis of the glucuronide conjugate during the warm acidic conditions prevailing in the Soxhlet apparatus. Failure to obtain glucuronide conjugate as such from the urine has now been shown to be due to the isolation procedure adopted as the glucuronide is strongly adsorbed on alumina from benzene and cannot be eluted with ethyl alcohol. It can be eluted, however, if a small amount of hydrochloric acid is added to the alcohol.

Although providing a satisfactory explanation for the final elimination of free 3-hydroxypyrene in both urine and faeces these experiments have yielded no evidence concerning the presence of dihydroxy compounds in the urine. No trace of these compounds or possible metabolic precursors has been obtained from the tissues examined so it is possible that they, like the quinones, are formed as secondary oxidation products from 3-hydroxypyrene. This problem is to receive further investigation.

SUMMARY

(1) Evidence has been obtained that the metabolic oxidation of pyrene to 3-hydroxypyrene proceeds via two intermediate stages.

(2) The experiments suggest that a primary metabolite, symbolised as PX, is formed in the liver and that this is converted into the glucuronide conjugate of 3-hydroxypyrene which is excreted in the bile and urine.

(3) During storage in the bladder or passage through the caecum and large intestine the glucuronide conjugate is then enzymatically hydrolysed to free phenol.

(4) The nature of the metabolite PX has not yet been determined.

### REFERENCES

HARPER, K. H.—(1957) *Brit. J. Cancer*, **11**, 499.

MARSH, C. A., ALEXANDER, F. AND LEVVY, G. A.—(1952) *Nature* (*Lond.*), **170**, 163.

WEIGERT, F. AND MOTTRAM, J. C.—(1946) *Cancer Res.*, **6**, 97.